# Hemochromatosis osteoarthritis

**DOI:** 10.3389/fendo.2026.1735750

**Published:** 2026-03-18

**Authors:** Yunze Yang, Zhenyue Zu, Yiran Huang

**Affiliations:** 1 Orthopedics and Traumatology, Heilongjiang University of Chinese Medicine, Harbin, Heilongjiang, China; 2 Puyang Traditional Chinese Medicine Hospital, Puyang, Henan, China; 3 Heilongjiang University of Chinese Medicine, Harbin, Heilongjiang, China

**Keywords:** hemochromatosis, osteoarthritis, iron overload, ferroptosis, therapeutic strategy, ferritinophagy

## Abstract

Hemochromatosis-associated arthropathy is a progressive degenerative joint disease characterized by iron-overload related pathological changes. While it may involve episodic inflammatory flares, it is primarily defined by bone and cartilage destruction rather than persistent chronic synovitis. It is typically classified as either spontaneous or secondary. This condition is often accompanied by various clinical manifestations, including liver disease, cardiomyopathy, skin pigmentation changes, diabetes mellitus, erectile dysfunction, and hypothyroidism. Diagnosis typically relies on a combination of imaging techniques such as x-ray, computed tomography (CT), magnetic resonance imaging (MRI), and ultrasound, along with iron function tests and joint fluid analysis. Genetic testing may also serve as an adjunctive diagnostic tool. The main treatment modalities currently available include phlebotomy (the primary therapy for hereditary hemochromatosis), iron chelators, joint cavity drug injections, and surgical interventions. Lastly, we delve into the preventative measures that can be adopted to mitigate the risk of developing this disease.

## 1 Introduction

Hemochromatosis osteoarthritis, a rare condition, stems from a decrement in the iron regulatory hormone hepcidin or a diminished binding capacity between hepcidin and ferroportin. This imbalance results in abnormal iron metabolism, causing systemic iron overload and subsequent deposition of hemosiderin and the presence of non-transferrin-bound iron (NTBI) within the synovial tissue and articular cartilage. These iron species promote the generation of reactive oxygen species (ROS) and exacerbate tissue damage. Notably, Ferroportin (FPN) serves as the sole known cellular iron efflux channel in mammals, and its activity is tightly regulated by the systemic iron regulatory hormone, hepcidin. While iron import is mediated by various proteins such as TfR1 and DMT1, the control of iron export via FPN is the critical determinant of systemic iron balance (1).

HFE is the gene encoding the human hemochromatosis protein, which maintains iron homeostasis by binding to transferrin receptor 1 (TfR1) and regulating the synthesis of hepcidin. Hereditary hemochromatosis is primarily caused by biallelic mutations in the HFE gene (e.g., p.C282Y), which result in the loss of HFE protein function. This dysfunction prevents stable binding to TfR1, subsequently leading to inappropriately low levels of hepcidin expression. Although hepcidin synthesis can still be partially stimulated by other pathways, such as the BMP6-SMAD signaling axis, the defect in HFE-mediated sensing prevents a sufficient hepcidin response to systemic iron overload. The ensuing increase in intestinal iron absorption and macrophage iron release leads to excessive iron deposition in tissues such as the liver and heart, ultimately causing organ damage. Additionally, a minority of cases are associated with mutations in genes such as HAMP (encoding hepcidin), HJV (encoding hemojuvelin), TFR2 (encoding transferrin receptor 2), and SLC40A1 (encoding ferroportin), all of which play a pivotal role in the regulation of systemic iron metabolism (2).

Hemochromatosis osteoarthritis is clinically divided into two categories: spontaneous and secondary. The spontaneous form primarily arises from abnormal iron metabolism stemming from genetic defects, particularly those involving the HFE gene variants on chromosomes, which have a strong association with enhanced iron absorption from food by the small intestinal mucosa (3). The secondary form of hemochromatosis osteoarthritis occurs when systemic iron overload deposits in the joints due to prolonged, massive, and repeated blood transfusions, hemolytic disorders, chronic liver disease, or extra-gastrointestinal iron supplementation (4–6).

Due to the accumulation of iron pigments in the joints, patients develop a distinct arthropathy, primarily affecting the wrist and ankle joints, with fewer occurrences involving the knee and hip joints (7). This characteristic arthropathy was first described in the metacarpophalangeal and proximal interphalangeal joints by Schumacher (8).

HFE gene mutations are closely related to iron - related diseases, and early genetic screening in high - risk populations is conducive to achieving early intervention and prevention of these diseases.

The aim of this article is to delve into the underlying mechanisms, diagnostic procedures, management strategies, and treatment options for hemochromatosis osteoarthritis. Furthermore, offer clinical guidance and assistance to physicians in the management of this condition.

## 2 Epidemiology

Mutations in the HFE gene, commonly referred to as hemochromatosis type 1, are typically observed in Caucasian individuals (9). These mutations primarily involve the substitution of guanine (G) for adenine (A) at nucleotide 845 of the HFE gene, leading to the replacement of cysteine with tyrosine at C282Y (10). Notably, despite the high frequency of C282Y homozygotes, only a subset of these individuals accumulate sufficient iron to cause tissue and organ damage, ultimately manifesting as arthritis.

The reported prevalence of joint diseases and joint pain in patients with hereditary hemochromatosis (HH) varies widely, ranging from approximately 28% to 81%. This broad interval reflects the heterogeneous nature of the disease and is heavily influenced by the criteria used for diagnosis (clinical *vs*. radiological) and the timing of study inclusion (11). Furthermore, while the incidence of p.Cys282Tyr homozygosity is nearly identical between males and females, significant disparities exist in the clinical prevalence and severity of arthropathy. This dimorphism is partly attributed to the protective effect of menstruation and pregnancy in females, which delays systemic iron accumulation, as well as potential gender-specific genetic modifiers that influence the penetrance of HFE mutations (2, 12).Recent longitudinal studies confirm that although joint symptoms may be the presenting feature in both genders, males often exhibit more advanced structural damage at the time of diagnosis (11).

The highest prevalence of C282Y homozygosity is observed in the Irish population (1.2%), with relatively high rates also noted among non-Hispanic white people (0.44%), Europeans (based on 27 studies with 6,302 samples, averaging 0.4%), and North Americans (0.5%). In contrast, the prevalence is significantly lower among Native Americans (0.11%), Hispanics (0.027%), Black people (0.014%), and individuals of Pacific Islander descent (0.012%). Asian individuals exhibit an extremely low prevalence (approximately 0.00004%), and no C282Y homozygotes were detected among 3,752 samples encompassing populations from Asia, the Indian subcontinent, Africa, the Middle East, and Australian Aboriginal groups (including indigenous peoples and Melanesians).

The frequency of C282Y heterozygosity is relatively high in European (9.2%) and North American (9%) populations, but ranges from only 0% to 0.5% in the aforementioned regions with low C282Y homozygosity prevalence. Regarding other genotypes, the prevalence of C282Y/H63D compound heterozygosity and H63D homozygosity is approximately 2% each in European populations, while in the Americas, these rates are 2.5% and 2.1%, respectively. Notably, non-Finnish Europeans have the highest C282Y allele frequency, reaching 5.14% (13–16).

In the United States, Europe, and Australia, the prevalence stands at approximately 1 in 200 to 400 individuals (17). Populations of Irish and Scandinavian descent exhibit the highest prevalence, while those of African descent have the lowest (18, 19). Variations in iron accumulation are attributed to factors such as living conditions, environmental differences, diet, and others.

## 3 Pathogenesis

### 3.1 Iron metabolism

The primary sources of iron in the human body are intestinal cells (responsible for dietary iron absorption) and macrophages (tasked with recycling iron from senescent red blood cells). Iron is released from these two types of cells into the plasma via the membrane protein ferroportin (FPN), which serves as the principal (and currently the only known) pathway for cellular iron release into the circulation in the human body (20, 21).

Hepcidin is a critical peptide hormone secreted by liver cells that centrally regulates systemic iron homeostasis by modulating the activity of ferroportin (22). Specifically, hepcidin binds to ferroportin, inducing its internalization and degradation, thereby inhibiting iron release from cells (such as intestinal cells and macrophages) into the plasma (23). This directly influences plasma iron levels, as well as the distribution and utilization of iron within the body.

### 3.2 Iron overload and joint

The precise pathophysiological mechanisms underlying the association between iron overload and bone and joint damage (particularly involving the synovium and cartilage) have not been fully elucidated. However, a growing body of clinical research evidence indicates a significant link between the two. Studies have demonstrated that in conditions of chronic iron overload, such as hereditary hemochromatosis and recurrent transfusion-dependent diseases, excessive iron is abnormally deposited in bone and joint tissues, especially in the synovium and articular cartilage. This iron deposition is believed to ultimately lead to joint structural damage and dysfunction (such as arthritis, cartilage degeneration, and osteoporosis) through pathways including the induction of oxidative stress, promotion of inflammatory responses, direct cytotoxic effects, and interference with bone and cartilage metabolism. This also partially explains why patients with chronic liver diseases (often accompanied by abnormalities in hepcidin synthesis or function) are prone to developing hemochromatosis-related bone and joint lesions.

As van Vulpen (24) observed, the mild hemorrhagic irritation caused by iron accumulation in joints can trigger an inflammatory response, ultimately contributing to the development of synovial inflammation. Conversely, Brighton’s animal experiments with rabbits revealed that injecting iron dextran induced only a minor synovial thickening, without eliciting an inflammatory cellular response (25). This indicates that synovial inflammation arises as a consequence of iron overload, which stimulates hemorrhage within the joint.

Carroll, G.J (26) demonstrated that in arthritis patients harboring mutations in the HFE gene, the concentration of ferritin in synovial fluid was significantly elevated, reaching 2 to 3 times higher levels compared to healthy individuals. This localized iron overload was found to directly or indirectly contribute to cartilage damage.

Heiland (27) uncovered a significant increase in neutrophil infiltration in cases of hemochromatosis-associated osteoarthritis, particularly in joint regions harboring iron deposits. This accumulation of neutrophils plays a pivotal role in the production of matrix enzymes, which expedite the degradation of cartilage and consequently contribute to heightened joint damage. Furthermore, Camacho (28) through his experiments on mice demonstrated that an elevated accumulation of iron in the synovial membrane of joints leads to cartilage degradation and ultimately the development of osteoarthritis.

Kai (29) underscored the significance of hereditary hemochromatosis (HH) in triggering synovial changes, cartilage degradation, and subchondral bone damage. Karim (30), on the other hand, delved into the antagonistic interplay between long-term iron accumulation in joints and the maintenance of cartilage homeostasis. In a two-year longitudinal observational study, Kennish (31) discovered a positive correlation between serum ferritin levels and the presence of osteoarthritis, as evidenced by radiographic imaging.

### 3.3 Mechanisms of iron overload-induced cartilage damage

Iron overload has the capacity to generate reactive oxygen species (ROS). These ROS inflict severe oxidative damage upon cell membranes, proteins, and DNA, thereby disrupting cellular architecture and function. Concurrently, ROS can activate signaling pathways, such as the nuclear factor kappa-light-chain-enhancer of activated B cells (NF-κB) pathway, thereby promoting the expression of inflammatory cytokines (e.g., interleukin-1β [IL-1β], tumor necrosis factor-α [TNF-α]) and matrix metalloproteinases (MMPs). This sets in motion a vicious cycle of inflammatory cascade reactions and cartilage matrix degradation (32).

On one front, iron accumulation can interfere with the expression and functionality of autophagy-related proteins, impeding the formation and degradation of autophagosomes. Consequently, damaged mitochondria are not promptly cleared and instead accumulate within cells, further exacerbating mitochondrial dysfunction and oxidative stress. On the other front, the aberrant autophagy process may activate inflammatory signaling pathways, facilitating the release of inflammatory cytokines and triggering synovial inflammation (33).

Immune cells activated by iron overload secrete cytokines and inflammatory mediators that directly target chondrocytes. For example, TNF-α and IL-1β not only inhibit the anabolic metabolism of chondrocytes, diminishing the synthesis of proteoglycans and collagen in the cartilage matrix, but also stimulate their catabolic metabolism and increase MMP secretion. Among these MMPs, MMP-13 serves as a pivotal enzyme for cartilage degradation, specifically targeting and degrading type II collagen, thereby compromising the structural and functional integrity of cartilage. Furthermore, the interaction between immune cells and chondrocytes can activate intracellular signaling pathways, such as NF-κB and mitogen-activated protein kinase (MAPK), further intensifying the inflammatory response and cartilage destruction (34, 35).

Iron overload drives the progression of inflammation and cartilage destruction by modulating the polarization and function of macrophages and T cells, regulating the expression of cell surface molecules and cytokine secretion patterns, and orchestrating interactions between immune cells and synovial cells as well as chondrocytes.

In summary, the precise pathogenesis of hemochromatosis osteoarthritis remains a scientific enigma, lacking a unified consensus. However, the cumulative findings from the aforementioned studies provide compelling evidence that they may be integral to the underlying processes. We eagerly await further rigorous research in the future to gain a more comprehensive understanding and achieve a unified perspective on the pathogenesis of this complex disorder.

## 4 Advanced mechanisms

### 4.1 Ferroptosis: a novel mechanism in hemochromatosis-associated osteoarthritis

Recent advancements in osteoarticular research have identified ferroptosis—an iron-dependent, non-apoptotic regulatory cell death characterized by lipid peroxidation—as a pivotal pathological driver in cartilage degeneration. In the context of hereditary hemochromatosis (HH), the joint microenvironment is subjected to chronic iron overload, providing a synergistic substrate for ferroptotic signaling pathways.

Multiple studies emphasize that ferroptosis in chondrocytes is triggered by diverse stimuli including mechanical overloading, inflammatory cytokines, and ionic flux. Wang et al. (36) demonstrated that excessive mechanical stress activates the Piezo1 channel, facilitating calcium influx and subsequent GPX4-regulated ferroptosis in chondrocytes. This suggests that in HH patients, whose joints are already compromised by iron deposition, mechanical irritation from daily activities may exacerbate ferroptotic damage via the Piezo1-calcium axis.

The molecular stability of Glutathione Peroxidase 4 (GPX4), the master antioxidant enzyme protecting cells from lipid peroxidation, is a central focus of recent therapeutic investigations. For instance, P21 has been shown to resist ferroptosis in osteoarthritic chondrocytes by enhancing GPX4 protein stability (37). In parallel, the inflammatory milieu characteristic of arthritic joints—specifically the presence of IL-1β and HIF-1α—further accelerates chondrocyte death. Chen et al. (38) revealed that inflammation-induced upregulation of TFRC (Transferrin Receptor) promotes the entry of ferrous iron (Fe^2+^) into chondrocytes, triggering the Fenton reaction and lipid peroxidation. This finding is particularly relevant to HH-related arthropathy, where systemic and local iron levels are inherently elevated, potentially creating a “vicious cycle” of TFRC-mediated iron uptake and ferroptotic destruction.

Furthermore, pharmacological interventions targeting the ferroptosis signaling pathway offer promising therapeutic avenues. Icariin (ICA) has been found to alleviate cartilage damage by enhancing the SLC7A11/GPX4 signaling pathway (39), while Spermidine exhibits protective effects against IL-1β-mediated ferroptosis (40). Given that current treatments for HH-associated OA are largely limited to phlebotomy and symptomatic relief, targeting the SLC7A11/GPX4 axis or utilizing ferroptosis inhibitors could represent a transformative approach to preventing cartilage loss in iron-overloaded joints.

### 4.2 The role of intracellular ferritin in chondrocyte damage

Intracellular iron homeostasis in chondrocytes is primarily managed by ferritin, which sequesters iron to prevent the formation of toxic free radicals. In the context of hemochromatosis, the mechanism of iron-induced damage is closely linked to the dysregulation of this protein. A key pathological process is ferritinophagy—the selective autophagic degradation of ferritin mediated by the cargo receptor NCOA4. This process triggers the abrupt release of stored iron into the labile iron pool (LIP), significantly increasing the intracellular concentration of redox-active ferrous iron.

This surge of free iron catalyzes the Fenton reaction, generating highly reactive hydroxyl radicals that lead to lipid peroxidation and the subsequent activation of the ferroptosis pathway. Moreover, excess iron from degraded ferritin accumulates in the mitochondria, disrupting the electron transport chain and inducing a burst of mitochondrial ROS (mROS). This synergistic organelle dysfunction promotes the expression of matrix metalloproteinases (MMPs), leading to the irreversible breakdown of the cartilage matrix.

### 4.3 Iysosome-iron-mitochondria axis

Beyond cytosolic lipid peroxidation mediated by the Fenton reaction, the disruption of iron homeostasis at the subcellular level plays a critical role in the pathogenesis of Hemochromatosis-associated osteoarthritis. Recent evidence suggests that dysregulation of the lysosome-iron-mitochondria axis is a key driver of pathological processes in bone-related conditions. In osteoclasts, lysosomal degradation of iron-containing proteins releases free iron ions, which are subsequently sequestered by mitochondria through mechanisms that remain to be fully elucidated. This influx interferes with mitochondrial oxidative phosphorylation and cellular energy metabolism (41). Similarly, in chondrocytes, aberrant iron trafficking leads to mitochondrial dysfunction and a burst of reactive oxygen species (ROS) production. This organelle-level oxidative stress synergistically interacts with the ferroptotic pathway, ultimately accelerating chondrocyte senescence and cartilage matrix degradation. These insights suggest that targeting subcellular iron homeostasis may provide a novel therapeutic orientation for the management of hemochromatosis-associated arthropathy.

## 5 Clinical manifestations

Hemochromatosis osteoarthritis typically manifests as a chronic and progressive monoarticular or polyarticular condition, exhibiting a unique clinical profile (42). Initially, patients may encounter mild joint discomfort or vague aches, symptoms that are often disregarded due to their subtlety and intermittent nature. However, as the disease progresses, symptoms such as joint pain, swelling, and restricted range of motion intensify, significantly impeding the patient’s daily activities and work capabilities (43).

In the progression of the disease, joint lesions assume a pivotal role. These lesions frequently initiate in the metacarpophalangeal and proximal interphalangeal joints, where mobility and weight-bearing capabilities are paramount for hand functionality. These joints typically manifest symmetrical lesions, affecting either single or multiple joints (44). As the condition advances, the lesions gradually spread to other joints, encompassing weight-bearing areas such as the hips, knees, and ankles. The damage to these joints can significantly compromise the patient’s mobility. Moreover, prolonged inflammation and damage may lead to the erosion of articular cartilage and bone, causing joint deformity, dysfunction, or even complete loss of joint function (45).

Beyond arthropathy, this disease can also trigger a spectrum of systemic symptoms. The liver, a crucial metabolic organ, is primarily affected, manifesting in asymptomatic elevations of serum aminotransferases, nonspecific right upper abdominal pain, hepatomegaly, or complications stemming from end-stage liver disease (46, 47). Although cardiac lesions are less common, with only 30 cases of dilated cardiomyopathy identified in a study of 3531 HH patients (48), the risk of cardiomyopathy resulting in patient mortality is significant (49).

Furthermore, this disease can culminate in diabetic complications as it can destroy pancreatic islet cells or disrupt insulin sensitivity (50, 51). Skin hyperpigmentation is another prevalent manifestation, stemming from the increased melanin production and deposition due to iron accumulation in the skin (52). Additionally, endocrine-related disorders, such as hypogonadotropic hypogonadism caused by iron deposition in the pituitary tract, are possible (53). Lastly, it may impair the immune system, associated with dysregulation of CD8+ T cells (54).

## 6 Diagnosis

Due to its insidious onset, the diagnosis of hemochromatosis osteoarthritis in its early stages is often overlooked or missed, as patients may initially only manifest mild general discomfort and a tendency for easy fatigue (55).

X-ray holds a pivotal position in the diagnosis of osteoarthropathy and serves as the preferred imaging modality for evaluating structural alterations in joints. For hemochromatosis-related osteoarthropathy, X-ray images exhibit a series of characteristic manifestations, particularly prominent in terms of bone erosion. X-ray films of patients with hemochromatosis-related osteoarthropathy often reveal bone erosion at the joint margins. This erosion typically initiates on the non-weight-bearing surfaces of the joints and gradually extends to the weight-bearing surfaces. At the sites of erosion, irregularities in the joint surface, discontinuity of the bone cortex, and even subchondral cystic changes can be observed. These alterations are particularly evident in the metacarpophalangeal and interphalangeal joints, representing typical manifestations of hemochromatosis-related osteoarthropathy.

As the disease progresses, the joint space gradually narrows due to the combined effects of cartilage destruction and bony hyperplasia. On X-ray films, uneven narrowing of the joint space can be clearly visualized, and in severe cases, joint ankylosis may even occur. Under the long-term stimulation of iron deposition, the subchondral bone plate undergoes sclerotic changes. On X-ray films, this is manifested as thickening and increased density of the subchondral bone plate, with irregular lucent areas sometimes visible, indicating the formation of subchondral cysts.

To cope with joint instability, the body forms bony hyperplasia at the joint margins to increase the contact area and stability of the joint. These hyperplastic changes appear as osteophyte formation around the joints on X-ray films, and in some cases, may even lead to the formation of loose bodies within the joint (56).

In hemochromatosis-related osteoarthropathy, X-ray films may also display some special manifestations, such as hooked bony protrusions. These protrusions typically occur at the head or base of the metacarpals, presenting in a hooked or beak-like shape, and represent one of the characteristic manifestations of hemochromatosis-related osteoarthropathy (57).

Ultrasound also holds significant value in the diagnosis of arthropathy associated with hemochromatosis. It can visualize synovial thickening, blood flow patterns, and changes in the periarticular soft tissues, providing auxiliary information for diagnosis. Specifically, ultrasound can detect hypoechoic areas caused by iron deposition within joints, as well as possible synovial hyperplasia and joint effusion. However, it should be noted that synovial hyperplasia is not a specific manifestation of hemochromatosis-related osteoarthropathy, as similar presentations can also be observed in other types of arthritis (such as rheumatoid arthritis). Therefore, in the clinical diagnostic process, a comprehensive analysis should be conducted by integrating the patient’s medical history, serum iron metabolism-related indicators (such as serum iron, ferritin, and transferrin saturation), and findings from other imaging examinations.

Regarding the differentiation between the synovitis of osteoarthropathy and inflammatory arthritis, ultrasound examination can offer some assistance but is not absolutely reliable.

The synovitis in osteoarthropathy is usually mild and is mostly related to joint degenerative changes and mechanical irritation; in contrast, the synovitis in inflammatory arthritis is more pronounced, often accompanied by a significant increase in blood flow signals and synovial thickening. Nevertheless, these manifestations are not absolute, and joint fluid analysis may be necessary for further definitive diagnosis when required (58).

CT plays an indispensable role in diagnosis, as it can precisely reveal the location of cartilage damage and accurately evaluate the bone details in the affected areas. This information is of great significance for assessing disease severity and formulating treatment plans (59).

MRI holds significant potential in offering noninvasive imaging biomarkers for hemochromatosis osteoarthritis. It empowers clinicians with crucial insights into the glycosaminoglycan content (via chemical exchange saturation transfer, sodium MRI, and T1ρ) and collagenous tissue status (including T2, apparent diffusion coefficient, and magnetization transfer) within joint structures, thus assisting in early-stage diagnosis (60). However, it should be clearly noted that although the deposition of hemosiderin in thickened synovium exhibits characteristic manifestations on MRI, it is not a specific indicator for the diagnosis of hemochromatosis. Some other diseases that cause intra-articular hemorrhage, such as hemophilic arthritis and traumatic intra-articular hemorrhage, may also lead to hemosiderin deposition in the synovium, resulting in similar hypointense changes on MRI. Therefore, in the clinical diagnostic process, a diagnosis of hemochromatosis cannot be made solely based on the manifestation of hemosiderin deposition in thickened synovium on MRI. A comprehensive analysis and judgment should be made by combining the patient’s medical history, serum iron metabolism-related indicators (such as serum iron, ferritin, and transferrin saturation), as well as other imaging examination results.

It can be distinguished from other forms of osteoarthritis through iron function tests and synovial fluid evaluations, encompassing screenings for serum iron, transferrin, serum ferritin levels, or the detection of iron deposits within the joint fluid (61).

Genetic testing, serving as a valuable adjunctive diagnostic tool, is routinely employed to screen for specific gene loci, including C282Y, H63D, and S65C, thereby assisting in the diagnosis. Screening for these loci is particularly useful in the early stages, prior to the emergence of clinical symptoms (10, 62).

## 7 Treatment

The prevention of hemochromatosis osteoarthropathy is paramount, with the fundamental strategy being the mitigation of iron accumulation and its progression, thereby averting further deterioration of osteoarthritis (29, 30). In instances where the disease is detected in its later stages or progresses rapidly, leading to a diagnosis of osteoarthritis, the treatment approach shifts towards conservative or surgical modalities. The overarching objective of treatment remains the alleviation of patients’ joint symptoms, while maintaining and enhancing their joint function to optimize their quality of life. Currently, the treatment recommendations for HH-OA are primarily based on evidence from primary osteoarthritis and expert consensus, necessitating further validation through high-quality research.

### 7.1 Pharmacologic treatment

#### 7.1.1 Iron chelators

Iron chelators have demonstrated remarkable effectiveness in the treatment of hemochromatosis, yet their clinical use in remains prevalent despite a paucity of therapeutic clinical data substantiating their efficacy. Currently, the approved chelators for marketing include deferoxamine, deferiprone, and deferasirox.

Desferrioxamine chelates with iron and aluminum ions, effectively reducing pathological deposits at the joints and inhibiting excessive collagenase-mediated type II collagen cleavage in cartilage, thereby reversing phenotypic changes (63, 64). However, it is crucial to be mindful of its toxic side effects with long-term use, including hypotension, renal insufficiency, neurotoxicity, growth retardation, and opportunistic infections, among which are the most serious (65).

Deferiprone plays a pivotal role in facilitating the intra- and extracellular transfer of iron. It efficiently directs iron from the extracellular milieu into the nucleus and mitochondria, while also aiding in the migration of iron between these two compartments. Furthermore, desferrioxamine induces the translocation of iron from endosomes to the nucleus and enhances the transport of iron from specific intracellular regions to the extracellular carrier transferrin. Notably, deferiprone mobilizes iron from iron-rich cells and provides it to pre-erythroid cells, supporting their hemoglobin synthesis process, regardless of the transferrin status (66, 67).

Deferasirox possesses the ability to bind to trivalent iron ions in a 2:1 ratio, resulting in a complex that enhances the elimination of iron from the body. Additionally, it competitively binds to heme synthase, hindering the decomposition of heme into ferrous ions, and further inhibits the binding of ferric ions to proteins. This reduces the formation of Fe-S helix-loop complexes, thereby mitigating iron overload, diminishing iron deposits in joints, and inhibiting the progression of osteoarthritis (68, 69). Furthermore, in animal models, deferasirox has demonstrated a capacity to limit joint inflammation and cartilage damage induced by blood (70).

While the three aforementioned oral medications effectively prevent and limit the onset and progression of osteoarthritis by chelating iron in the body, the side effects of iron chelators cannot be overlooked. Moreover, in cases of more severe osteoarthritis symptoms, the sole use of iron chelators often yields limited therapeutic benefits and a relatively small reversal effect. Therefore, to achieve more effective treatment of osteoarthritis, it is generally advisable to combine these medications with other therapeutic drugs or methods to enhance their efficacy and ultimately improve the quality of life for patients.

#### 7.1.2 Nonsteroidal anti-inflammatory drugs

NSAIDs are commonly employed as a first-line approach, with proven efficacy and safety, for managing localized osteoarticular pain and serving as an adjunctive therapy alongside other modalities (71). When considering therapeutic options, topical NSAIDs are often prioritized due to their comparable effectiveness to oral formulations while exhibiting significant advantages in mitigating gastrointestinal reactions and reducing cardiovascular risks (72). Among topical NSAIDs, diclofenac diethylamide and etofenproxil are typically preferred as choice drugs. If oral NSAIDs, including cyclooxygenase (COX)-2 inhibitors, are deemed necessary, it is advisable to administer the lowest effective daily dose to minimize gastrointestinal, renal, and cardiovascular side effects, which are often dose-dependent (73).

#### 7.1.3 Antioxidants

In recent years, a large number of experiments have demonstrated that antioxidants can significantly inhibit the expression of catabolic markers related to iron-overload-induced osteoarthritis and mitigate mitochondrial dysfunction, thereby slowing down the process of cartilage degeneration (74). Specifically, N-Acetylcysteine (NAC) mitigates oxidative stress, safeguards osteoclasts from apoptosis caused by iron overload, maintains proteoglycan content, decreases histological disease scores, and normalizes the metabolic functions of chondrocytes (75). Furthermore, *in vitro* studies have demonstrated that NAC safeguards chondrocytes from interleukin 1-induced oxidative stress, thereby reducing chondrocyte apoptosis and degeneration (76).

#### 7.1.4 Opiates

The utilization of opioids for pain management in osteoarthritis stemming from iron overload is generally discouraged due to the high incidence of adverse effects and the potential for opioid addiction (77). In a thorough examination of patients with osteoarthritis, we discovered that the administration of tramadol as a therapeutic option significantly elevated the risk of all-cause mortality among patients (78).

### 7.2 Bloodletting

Phlebotomy has been practiced for over 3,000 years. Although it faced skepticism in the 19th century, it remains a primary treatment approach for primary hemochromatosis to this day. In contrast, iron chelation therapy is more commonly employed for the treatment of secondary hemochromatosis (79). Generally, during the initial treatment phase, the frequency of phlebotomy may be relatively high, such as once a week, with 400–500 ml of blood being drawn each time. As the patient’s condition improves, the frequency of phlebotomy is gradually reduced until the serum ferritin level is maintained at around 50 μg/L to prevent disease recurrence (80). Although phlebotomy can reduce iron levels in the body, its direct therapeutic effect on osteoarthritis has not been clearly confirmed.

### 7.3 Joint cavity injections

Hyaluronic acid (HA), a naturally occurring polysaccharide abundantly present in the human body, is endogenously produced by the body and possesses unique physicochemical and biological properties. Its excellent biocompatibility and biodegradability make it one of the most frequently employed substances for intra-articular injections (81). HA effectively reduces the migration of inflammatory factors, enhances synovial fluid within the articular cavity, and safeguards the cartilage to minimize friction (82). Notably, the combined application of platelet-rich plasma (PRP) and corticosteroids significantly outperforms the use of HA alone in intra-articular injections, significantly alleviating pain and enhancing functional outcomes in patients (83, 84).

In recent years, PRP has gradually become one of the treatment modalities favored by clinicians due to its remarkable therapeutic efficacy. By delivering a supraphysiological concentration of autologous platelets to the site of tissue injury, PRP enhances the healing of bones and soft tissues (85). Compared with HA and corticosteroids, intra-articular injections of PRP demonstrate superior long-term outcomes in terms of pain reduction and functional improvement for patients (86–89). However, further exploration is warranted owing to the relatively high cost of PRP and significant inter-individual variability in treatment responses.

Corticosteroids exhibit a swift onset of action in alleviating acute pain in osteoarthritic joints, providing notable short-term benefits. However, their efficacy in providing long-term pain relief and functional improvement is limited (90). Repeated administrations of corticosteroids have been shown to accelerate cartilage loss and suppress protein expression, thus their use should be minimized unless deemed necessary (91).

### 7.4 Surgical treatment

Surgical interventions for joint disorders typically encompass articular cartilage repair, arthroscopic surgical techniques, and joint replacement.

Arthrochondral repair surgery employs surgical intervention combined with tissue engineering strategies to repair injuries to the hyaline cartilage on the joint surface (92). However, special attention must be paid to the fact that this surgical approach falls within the contraindicated scope for the treatment of primary osteoarthritis. Particularly when it comes to hemochromatosis-related osteoarthritis, its characteristics of multi-joint involvement, the pathological progression of persistent cartilage degradation, coupled with the clinical feature of patients generally having an advanced age at onset, render this therapeutic strategy an absolute contraindication for joint lesions associated with hemochromatosis.

Arthroscopic surgery effectively diminishes inflammatory factors in the synovial fluid of patients’ joints, thereby improving joint pain and functionality (93). Nevertheless, there is a dearth of arthroscopic literature pertaining to hemochromatosis osteoarthritis, rendering the treatment approach and prognosis following arthroscopic surgery uncertain.

Joint arthroplasty should be considered for patients with extreme limitation or loss of joint function, when other treatment modalities fail to provide significant relief. The decision should be made jointly with the patient, taking into account their age, physical condition, and other relevant factors.

For lesions affecting cumulative small joints, orthotic devices can be considered to alleviate symptoms to a certain extent by adjusting the biomechanical environment and restricting abnormal joint movements. However, if effective intervention targeting the primary cause is not carried out, as the disease progresses, the pathological changes in the joints will continue to worsen. At this point, the biomechanical regulatory effects of the orthotic devices will gradually diminish, making it difficult to achieve long-term and stable therapeutic outcomes.

### 7.5 Physiotherapy

#### 7.5.1 Exercise therapy

Low-impact aerobic exercises such as swimming, cycling, and elliptical training can improve joint range of motion, enhance muscle strength, and reduce joint loading. Studies have shown that such exercises can significantly alleviate pain and improve function in patients with HOA (94). Light-to-moderate resistance training (e.g., elastic band exercises) can enhance the stability of muscles surrounding the joints, delay cartilage degeneration, improve physical performance, and reduce anxiety and depression (95). Balance and coordination training: Practices such as Tai Chi and Pilates can improve core stability and proprioception, thereby reducing the risk of falls.

#### 7.5.2 Manual therapy

For joint stiffness in HOA patients, manual therapy can improve joint range of motion. However, excessive manipulation should be avoided to prevent further damage to iron-deposited tissues. Soft tissue massage, combined with heat or cold therapy, can relieve muscle spasms, but it should be integrated with exercise therapy to maintain long-term effects (96).

#### 7.5.3 Physical agent modalities

Transcutaneous Electrical Nerve Stimulation (TENS): Short-term use of TENS can relieve acute pain, but long-term efficacy requires combination with exercise (97). Cold therapy (e.g., ice application) during the acute phase can reduce inflammation, while heat therapy (e.g., paraffin wax therapy, Photodynamic treatment) during the chronic phase promotes blood circulation. However, high temperatures should be avoided to prevent increased oxidative stress in iron-deposited areas (98).

Due to iron deposition leading to fragile joint cartilage, HOA patients should avoid high-impact exercises (e.g., running, jumping) and are recommended to engage in non-weight-bearing exercises (e.g., hydrotherapy). Physiotherapy should be combined with phlebotomy or iron chelators to reduce the continuous damage caused by iron overload to the joints. Currently, there are limited studies specifically targeting HOA, with most evidence derived from patients with general OA. Future cohort studies on HOA are needed to explore individual differences in response to physiotherapy due to iron deposition.

The treatment strategy should be systematically planned based on disease control requirements: First, implement intervention measures including bloodletting therapy, rational use of iron chelators, and genetic screening. Subsequently, carry out conservative treatment, which encompasses strictly limiting iron intake, avoiding the use of vitamin C, emphasizing joint protection, and appropriately administering analgesics. If the disease progresses to a stage requiring surgical intervention, adopt arthroscopic treatment up to joint replacement surgery for large joint lesions, and utilize arthroscopic treatment up to arthrodesis for small joint lesions.

For specific procedures, please refer to the treatment flowchart shown in **Table 1**.

**Table 1:** Summary of therapeutic strategies for hemochromatosis-associated arthropathy.

Category	Treatment modality	Mechanism/Goal	Key considerations & limitations
Systemic Iron Control	Therapeutic Phlebotomy	Reduces systemic iron stores by removing excess iron.	First-line therapy; Target: Serum ferritin < 50–100 μg/L; early initiation may delay arthropathy progression.
	Iron Chelation	Promotes iron excretion via chelating agents (e.g., Deferoxamine).	Indicated for patients contraindicated for or intolerant to phlebotomy; monitor for adverse effects.
Pharmacological Management	Analgesics (e.g., Acetaminophen)	Symptomatic relief of mild to moderate pain.	Used as needed; monitor for hepatotoxicity, particularly in iron-overload patients.
	NSAIDs	Anti-inflammatory and analgesic effects.	Effective for managing acute inflammatory flares; caution regarding gastric and renal toxicity.
	Intra-articular Injections	Corticosteroids or Hyaluronic acid.	Localized potent anti-inflammatory effect; typically limited to 3–4 injections per year.
Emerging Targeted Therapy	Ferroptosis Inhibitors*	Enhancing SLC7A11/GPX4 pathway (e.g., Icariin, Spermidine).	Currently in experimental stages; represents a potential direction based on novel mechanisms.
Non-Pharmacological Therapy	Physiotherapy	Maintain joint mobility and strengthen periarticular muscles.	Recommend low-impact exercises (swimming, cycling); avoid high-impact activities (running, jumping).
	Lifestyle Modification	Reducing mechanical load and oxidative stress.	Weight management is crucial; avoid high-temperature environments to minimize oxidative stress.
Surgical Intervention	Total Joint Replacement (TJR)	Reconstruction of joint function and elimination of pain.	Indicated for end-stage arthropathy; systemic iron control must continue post-surgery.
	Arthrodesis	Provides joint stability and pain relief.	Often used as a salvage procedure for specific joints (e.g., the ankle).

*Represents potential therapeutic strategies based on cutting-edge mechanistic research; not yet widely implemented in clinical practice.

## 8 Prevention

We should actively carry out publicity and education efforts to enhance the public’s awareness of hemochromatosis-related osteoarthritis. Admittedly, the prevention of osteoarthritis requires multidimensional and comprehensive interventions. However, for hemochromatosis-related osteoarthritis, public awareness campaigns play an irreplaceable and pivotal role in promoting early screening and iron metabolism management. In high-incidence areas and among individuals with a family history of the disease, regular iron function tests should be conducted to closely monitor dynamic changes in iron levels within the body. In this way, the disease can be detected early and treated promptly, thereby effectively controlling its progression and preventing further deterioration. Dietary considerations are also crucial; individuals should avoid excessive intake of iron and vitamin C. Vitamin C is a potent antioxidant that neutralizes free radicals in the body, reduces oxidative stress-induced cellular damage, and helps protect the health of cells and tissues. Additionally, vitamin C plays a vital role in collagen synthesis, which is essential for maintaining the normal structure and function of tissues such as the skin, bones, and joints. However, vitamin C intake can promote iron absorption, exacerbating iron overload in the body and causing more severe damage to vital organs such as the liver and heart. To reduce the risk of the disease, people should minimize the use of iron utensils and cut down on the consumption of animal organs in daily life.

For asymptomatic carriers or high-risk family members, this study offers the following specific preventive recommendations: Regular screening for related diseases (such as serum ferritin testing and genetic testing) should be carried out to identify potential risks early and initiate interventions; individuals should actively adjust their lifestyle, including maintaining a balanced diet, engaging in regular exercise, quitting smoking, and limiting alcohol consumption, with particular emphasis on avoiding factors that may increase disease risk; meanwhile, individuals with a family history of genetic disorders are advised to seek professional genetic counseling to understand disease risks and mechanisms and develop personalized scientific prevention plans.

## 9 Conclusion

Hemochromatosis osteoarthritis, as a rare chronic disease, is often prone to misdiagnosis in its early stages due to its inconspicuous symptoms and can even be confused with other types of arthritis. In the initial phase of the disease, patients may only experience mild discomfort. However, over time, it can gradually progress to severe pain in multiple joints. In severe cases, it may even lead to complete loss of joint function, causing significant distress in patients’ daily lives.

For the early diagnosis of hemochromatosis osteoarthritis, a combination of multiple screening methods is usually required, such as imaging examinations and hematological analyses. Meanwhile, genetic testing, as an advanced diagnostic approach, also provides strong support for the accurate diagnosis of this disease. These comprehensive detection methods can assist doctors in more accurately assessing patients’ conditions and provide a solid basis for subsequent treatment.

In terms of treatment, targeting the primary cause of hemochromatosis osteoarthritis, doctors typically use iron chelators to reduce free iron in the joints, thereby lowering the inflammatory response within the joints. Additionally, based on patients’ pain symptoms and impaired joint function, doctors will formulate personalized treatment plans, including medication, physical therapy, and rehabilitation exercises, to alleviate patients’ suffering to the greatest extent and restore their joint function.

From a clinical perspective, a major unresolved issue pertains to the long - term effectiveness and safety of current treatment approaches for diseases associated with HFE gene mutations. For instance, although phlebotomy is a well - established method for treating iron overload, its long - term impact on patients’ quality of life, particularly in terms of fatigue and cognitive function, remains inadequately understood. Moreover, there is still controversy regarding the optimal timing to initiate treatment for asymptomatic carriers of HFE gene mutations. Some studies recommend early intervention, while others advocate for a watch - and - wait strategy, and more research is needed to clarify this matter.

In the research field, although we have clearly established a close association between HFE gene mutations and hemochromatosis osteoarthritis, there are still many unknowns regarding the specific molecular mechanisms by which these gene mutations trigger the disease. Delving deeper into these mechanisms holds the promise of providing a theoretical basis for the development of more precise and effective targeted therapeutic drugs. Additionally, the search for more sensitive and specific biomarkers for early diagnosis to enable timely intervention and treatment at the early stage of the disease is also a crucial direction for future research.

However, it is worth noting that there is currently no cure for hemochromatosis osteoarthritis. This means that patients have to face the challenges posed by the disease over the long term and receive continuous treatment and management. Nevertheless, we still look forward to more research and innovations in the future that can bring more effective treatment methods to patients with hemochromatosis osteoarthritis, enabling them to get rid of the disease’s troubles as soon as possible and regain a healthy life.
